# Selective Recovery of Zinc from Metallurgical Waste Materials from Processing Zinc and Lead Ores

**DOI:** 10.3390/molecules24122275

**Published:** 2019-06-19

**Authors:** Wojciech Hyk, Konrad Kitka, Dariusz Rudnicki

**Affiliations:** 1 Faculty of Chemistry, University of Warsaw, Pasteura 1, PL-02-093 Warsaw, Poland; 2 Faculty of Chemistry, Biological and Chemical Research Center, University of Warsaw, Żwirki i Wigury 101, PL-02-089 Warsaw, Poland; 3 Greenmet Technology Ltd., Chełmońskiego 67, PL-96-321 Żelechów, Poland; darek.greenmet@gmail.com

**Keywords:** zinc, zinc ferrite, leaching, metallurgical waste materials, stockpiles

## Abstract

A method for processing of metallurgical waste materials (chemically defined as sulfur-bearing zinc-ferric materials) produced by plants processing zinc ores and their concentrates is proposed. The method proposed is a combination of pyro- and hydrometallurgical treatments of the waste material. The crucial steps in the developed method include: roasting the material at 450 °C to generate sulfur dioxide (SO_2_), absorption of SO_2_ in an aqueous system to form sulfuric acid (IV), carbothermic decomposition of zinc ferrite compounds, and leaching of zinc from the roasted material using sulfuric (IV) acid. The method allows one to extract up to 40% of zinc from the waste material and, consequently, to generate a fraction of material with substantially higher content of iron oxides. The proposed method takes advantage of the presence of sulfur in the processed material which upon roasting is converted to sulfuric acid (IV)—a leaching agent for selective extraction of zinc. The properly adjusted pH of the aqueous medium in which the leaching process is carried out is the key factor determining the quantitative and selective separation of zinc. If the amount of sulfur in the processed material is insufficient, it may be supplemented by adding sulfuric acid (VI) to adjust the pH. The method proposed was tested at a laboratory scale and quarter industrial scale using the real samples taken from stockpiles in the vicinity of the plant processing zinc and lead ores in Poland. It may also work for any zinc-ferric materials from various sources.

## 1. Introduction

Zinc sulfide ores or their concentrates are the major primary sources of zinc [1]. Processing of these sources results in the production of residues containing metallurgical slags which are frequently stored in waste piles or landfills. From a chemical point of view, these waste materials are rather complex compositions of oxides, sulfides, carbonates and silicates of metals, whose approximate percentages by weight are within the following ranges: iron—15% to 28%, zinc—3% to 12%, calcium compounds—up to 16%, silica—up to 12%, sulfur—up to 9%, lead—0.5% to 6%, copper and arsenic—up to 2%. Zinc present in the material is mostly chemically bonded with iron and non-metallic elements and forms a series of compounds [1] that can be referred to as zinc-ferric compounds. Zinc ferrite is the one of the most abundant representatives of this class of compounds.

Recently, there is an increasing interest in use of zinc slag-containing materials as partial substitutes for sand in road materials. However, this area of applications of metallurgical waste materials requires detailed knowledge on their long-term behavior and eco-compatibility [2]. On the other hand, relatively high metal content makes these materials valuable potential secondary sources of zinc and iron. Their processing (additionally motivated by environmental significance [3]) requires development of methods for the economic extraction of the crucial metals. The key aspect in these treatments is the chemical destruction of the ferrite structure. This task can be achieved hydrometallurgically using highly aggressive media at increased temperature. Zinc ferrite decomposition may involve such leaching reagents as: iron (III) chloride or concentrated sulfuric (VI) acid. The reaction with iron (III) chloride at a temperature higher than 100 °C consists of O^2−^/Cl^−^ exchange allowing the recovery of zinc as ZnCl_2_ and iron as Fe_2_O_3_ [4]. The dissolution of zinc ferrites in hot and highly concentrated sulfuric (VI) acid results in the production of zinc and iron sulfates (VI). The efficiency of zinc leaching from a prepared material has been analyzed under varying conditions [5]. The employment of the highly aggressive media makes the proposed approaches less practical for the application at a larger scale.

A pyrometallurgical method for the decomposition of zinc ferrite was employed for electric arc furnace (EAF) dust generated in a steel production [6,7]. It has been shown that zinc ferrite can be efficiently decomposed by heating the dust with an excess of CaO at 900–1100 °C. The formed ZnO was then separated from ferrites by applying a strong magnetic field.

The data published in the literature may suggest that the best performance of processing of waste materials can be achieved by combing pyro- and hydrometallurgical methods [1]. The presence of ferrites gives the zinc slag-containing materials, in which they are present, magnetic properties, but their complex chemical (intermetallic connections) form prevents simple separation of iron compounds from zinc compounds via an external magnetic field.

In our work a novel combination of pyro- and hydrometallurgical methods for the treatment of waste materials from processing of zinc and lead ores is presented. The thermal and chemical stages of the developed procedure are supported by a magnetic field. Magnetic separation reduces amounts of the chemicals used for the targeted treatment of the zinc-containing fraction. The developed methodology allows one to leach zinc selectively and to form an aqueous solution of zinc salt, leaving out other major metallic constituents present in the material, i.e., iron and lead. The essential idea of the proposed approach is based on a two-step roasting process of the zinc-ferric materials and on employment of the generated gaseous sulfur dioxide (SO_2_) for the zinc leaching process. Sulfur dioxide dissolved in a suitable amount of water is a factor that enables selective conversion of zinc compounds to aqueous soluble forms. The presence of bonded sulfur in the form of metal sulfides in waste materials gives the ability for easy and cost-effective generation of SO_2_ by roasting in a chemically non-aggressive environment. The process of selective recovery of zinc does not require any additional raw materials. The only elements needed are energy for heating and water which provides an environment for dissolution of zinc compounds.

The method developed was tested at a laboratory scale and quarter industrial scale using the real samples taken from stockpiles in the vicinity of the plant processing zinc and lead ores in Poland. The proposed approach can work for any zinc-ferric materials from various sources such as steelmaking flue dusts, leach residues, and secondary materials of similar nature.

## 2. Materials and Methods

### 2.1. Chemicals

Sulfuric (VI) acid (H_2_SO_4_) and sodium carbonate (Na_2_CO_3_) used for the processing of test samples were of analytical grade and were purchased from POCH (Gliwice, Poland). All solutions were prepared using high purity water obtained from a Milli-Q Plus/Millipore purification system.

### 2.2. Instrumentation

Grinding and magnetic separation of the prepared samples were performed using a laboratory ball mill and a drum magnetic separator designed and assembled by Technical Center of Eko-Lab (Poland). The separator consists mainly of a permanent magnet system, a rotating drum, a separating chute, a feed tank, and (optionally) a flushing pipe. The magnetic field intensity on the surface of the drum can vary between 0.1 and 0.8 T. The sample enters the separator through the separating chute, magnetic particles from the sample are attracted by magnetic forces to the surface of the drum, and non-magnetic fraction falls down by gravity or water flow (if wet separation is employed). The magnetic fraction is then transferred by the rotating drum to the top where the magnetic field is close to zero, and it is then mechanically removed from the drum.

The roasting processes were conducted in either a flame heated tube or a rotary kiln at a larger scale (provided by the Institute of Ceramics and Building Materials (Kraków, Poland)).

An X-ray Fluorescence Spectrometer (model S1 Titan, Bruker, Billerica, MA, USA) (XRF) was used for determinations of the chemical composition of the material examined.

An Inductively Coupled Plasma Mass Spectrometer (NexION 300D, PerkinElmer, Waltham, MA, USA) (ICP-MS) was used for the validation of the results produced by the XRF method.

An X-ray Powder Diffractometer (model D8 Discover, Bruker) (XRPD) was employed for the polycrystalline structural analysis of the homogenized waste material. A diffraction angle of 10 to 70 deg was scanned at a rate of 5 deg/min.

pH values of aqueous solutions were determined by a portable Methrom pH-meter.

### 2.3. Sampling and Sample Pretreatment

Three sections of stockpiled waste materials from processing zinc and lead ores, located in the vicinity of Zn-Pb mining and smelting complex in Poland, were selected. Each section of the area of approximately 100 m^2^ was randomly sampled. Samples of waste materials were taken at different locations and depths (up to 5 m). All samples were averaged by mixing and the overall sample was formed.

The overall samples from each section of stockpiled waste materials were gradually sieved and homogenized by grinding them in a ball mill (grain diameter—less than 0.1 mm). Test samples of 1 kg were further employed in laboratory experiments.

### 2.4. Chemical Analysis

To obtain reliable and unbiased analytical results by using the XRF technique it is extremely important to prepare properly the solid sample and to perform method validation. The metallurgical samples were homogenized by grinding them in the ball mill to obtain fine powders of a grain diameter less than 0.1 mm. This step was especially vital for reliable results since the radiation intensity that reaches the sample volume element is attenuated in traveling a given pathlength. The XRF method was validated by comparing the analytical results produced by our XRF setup with those provided by a certificate of the reference material (the standard geological sample SAR-M, Bruker). Two major components of the measurement uncertainty, i.e., random (expressed by the measurement repeatability and given by relative standard deviation *RSD_rep_* = 0.082) and systematic (quantified by the recovery factor, *R* = 93%, and bias, Δ = –0.064% (*m*/*m*)), were evaluated on the basis of replicate measurements for the selected real and the standard samples. The validation characteristics of the XRF method allowed us to estimate the combined uncertainty associated with the element determinations. The average value of the relative combined uncertainty for the XRF determinations in the samples examined was estimated to be 9%. The results produced by the proposed XRF method were consistent within the expanded uncertainty (at 95% confidence) to those obtained by ICP-MS method which required mineralization of the sample prior the measurements.

Each 1 kg sample was analyzed three times. Chemical compositions of the materials examined are listed in Table 1.

### 2.5. The Procedure of Zn Recovery from Zinc-Ferric Metallurgical Waste Materials

The proposed procedure consisted of the following steps:

I) treatment of the powdered zinc-ferric material with an external magnetic field;
II) roasting of the “non magnetic” fraction at a temperature of 450 °C with full air access;
III) absorption of gaseous SO_2_ by water at 25 °C;
IV) carbothermic decomposition of zinc ferrite at temperature 750 ± 50 °C;
V) leaching of zinc from the roasted material using sulfuric (IV) acid with an optional addition of sulfuric (VI) acid;
VI) neutralization of the leaching solution with a saturated aqueous solution of sodium carbonate;
VII) wet magnetic separation of the leaching residue.

**Figure 1:**
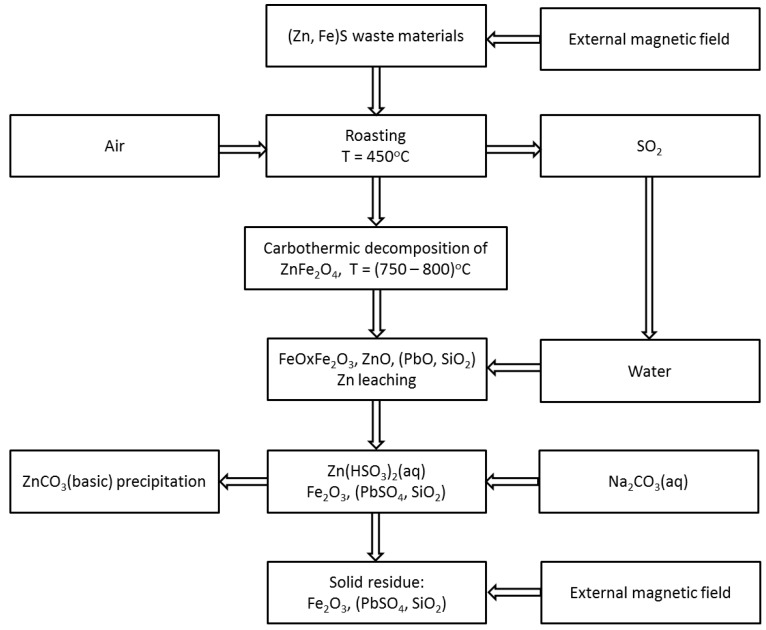
Scheme of the method for processing zinc-ferric waste materials.

**Figure 2:**
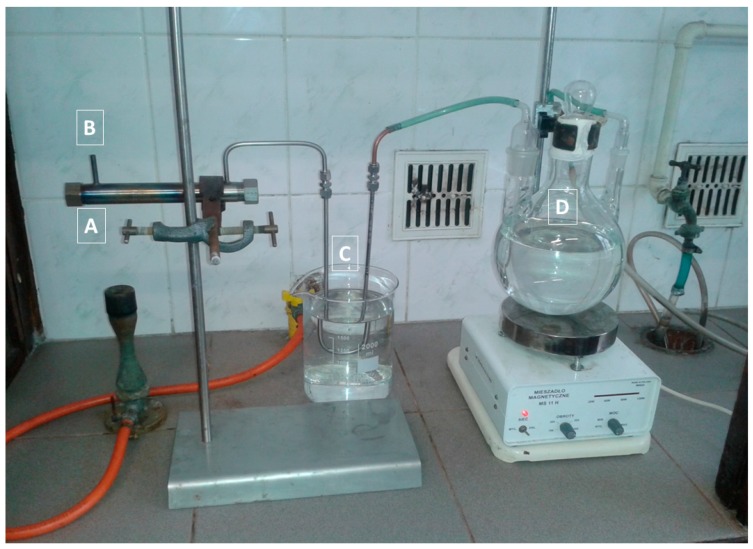
The instrumentation designed for laboratory tests of the method of processing zinc-ferric waste materials: furnace (**A**) for roasting and carbothermic decomposition of zinc ferrite, air access (**B**), heat exchanger (**C**), SO_2_ water collector (**D**)—production of leaching agent.

The procedure worked out is illustrated schematically in Figure 1. The equipment set up for laboratory tests is presented in Figure 2.

## 3. Results and Discussion

The proposed mechanism of the zinc extraction from zinc-ferric wastes involves the following physical and chemical transformation steps:

I) Treatment of the zinc-ferric material with an external magnetic field—removal of the ferromagnetic fraction using a drum magnetic separator. The dry magnetic separation process produces two fractions: ferromagnetic (mainly consisted of iron oxides) and the one with a negligible response to the magnetic field (“non magnetic” fraction) containing sulfur-bearing zinc-ferric material.
II) Roasting of the “non magnetic” fraction at a temperature of 450 °C with full air access—decomposition of sulfide forms of metals and generation of gaseous SO_2_. The thermal decomposition of sulfur-bearing zinc-ferric material can be represented by the following general chemical equation:
  ZnFe_2_S_4_ + xO_2_*→* (Zn,Fe)(S,O) + ySO_2_, (1)
  where ZnFe_2_S_4_ is a representative of the group of zinc iron sulfides of the general formula (Zn,Fe)S and of various stoichiometries and mineralogical origins, and (Zn,Fe)(S,O) represents a class of oxidized forms of zinc and iron. Depending on the iron content, the crystallographic structures of (Zn,Fe)S compounds may be closely related to the structures of the typical zinc minerals like sphalerite, wurtesite, or marmatite. It is worth noting that the trials performed in a rotary kiln at a larger scale were also inspected by a unit of Inspectorate of Environmental Protection. The analysis of the composition of gaseous products performed by using gas analyzers revealed the presence of SO_2_ accompanied by negligible amounts of NO_x_ products. No other (organic or inorganic) pollutants were detected.
III) Absorption of gaseous SO_2_ by water at 25 °C—formation of sulfuric (IV) acid (leaching agent) according to the equilibrium reaction:
  (2) $${\mathrm{SO}}_{2}\;+\;H_{2}O\;⇄\;H^{+}\;+\;{{\mathrm{HSO}}_{3}}^{-}.$$
IV) Carbothermic decomposition of zinc ferrite [8,9] at temperature of 750 ± 50 °C using a flame heated tube (or a rotary kiln at a larger scale). The increase in temperature up to 750 °C favors formation of zinc ferrites which decompose under the reducing conditions according to the following chemical equation:
  3ZnFe_2_O_4_ + C *→* 3ZnO + 2Fe_3_O_4_ + CO. (3)
V) Leaching of zinc from the roasted material using sulfuric (IV) acid with an optional addition of sulfuric (VI) acid—formation of well soluble metal bisulfate (IV) as described by the following chemical reaction:
  ZnO + H_2_O + 2SO_2_*→* Zn^2+^ + 2HSO_3_^−^. (4)
VI) Neutralization of the leaching solution with a saturated aqueous solution of sodium carbonate (Na_2_CO_3_)—precipitation of zinc in the form of zinc carbonate
  (5) $${\mathrm{Zn}}^{2+}\;+\;{{\mathrm{CO}}_{3}}^{2-}\;⇄\;{\mathrm{ZnCO}}_{3}.$$
VII) Thickening of iron content by wet magnetic separation of the leaching residue.

**Figure 3:**
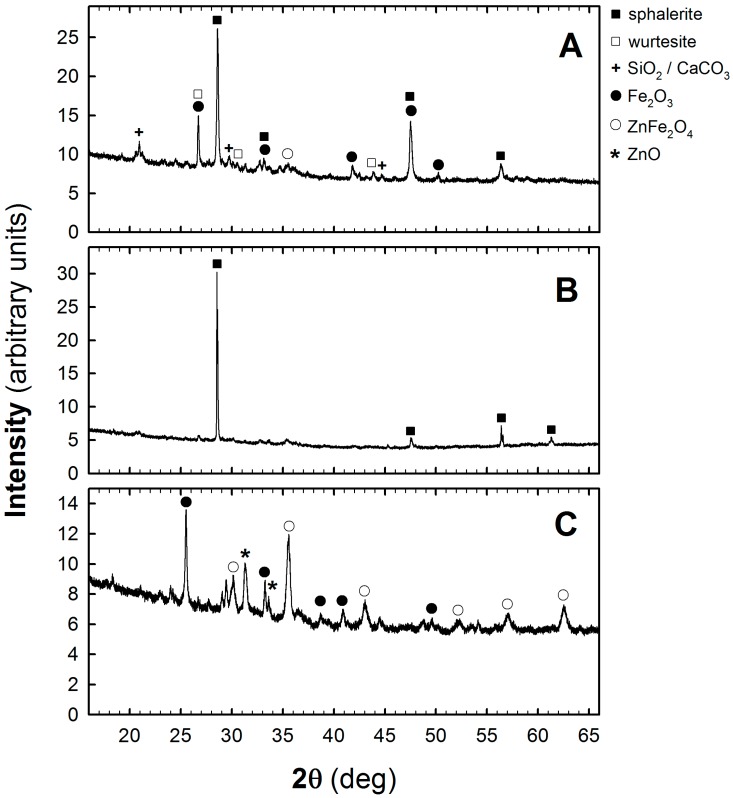
X-ray Powder Diffractometer (XRPD) patterns of zinc-ferric material after its homogenization (**A**), magnetic separation (**B**), and roasting processes (**C**).

An exemplary set of XRPD patterns were recorded for the test sample no. 1 (see Table 1) after its homogenization, magnetic separation, and roasting process, as shown in Figure 3. The XRPD patterns labelled with letters A, B, and C refer to the initial sample, “non magnetic fraction” (stage I of the processing method), and the residue obtained after the roasting process is completed (stages II–IV), respectively. As it seen in Figure 3A sphalerite is a dominant phase [10] in the zinc-ferric materials. In addition to sphalerite other phases such as wurtesite, quartz, and iron (III) oxide [11] can also be seen. After magnetic separation the peaks related to Fe_2_O_3_ phase disappear, as is observed in Figure 3B. Sphalerite becomes practically the only phase in the processed material. The roasting process results in a decomposition of sulfur-bearing zinc-ferric material and a formation of gaseous SO_2_. The further increase in temperature to 750 °C favors the formation of ZnFe_2_O_4_ which, under the reducing conditions, is then converted to ZnO and Fe_2_O_3_ phases. The XRPD pattern of the sample at this stage of material processing, presented in Figure 3C, shows dominating peaks around 2θ = 35.5, 32, and 25.5 that correspond to the presence of ZnFe_2_O_4_ [12], ZnO [12] and Fe_2_O_3_ [11], respectively.

The first stage of the roasting process at 450 °C was carried out as long as sulfur dioxide is generated, or pH of the resulting solution reaches the value of 2. The temperature of water used for this purpose should not exceed 25 °C so as not to limit the solubility of SO_2_ via elevated temperature. The next stage of the roasting process was carried out for at least 1 h in an increased temperature (750–800 °C) and requires addition of up to 10% (*m*/*m*) of carbon (charcoal). At this temperature carbothermic decomposition of zinc ferrite occurs.

An alternative way for the zinc ferrite decomposition may involve concentrated and hot sulfuric (VI) acid. According to our studies at least 33% (*m*/*m*) sulfuric (VI) acid at 75 °C needs to employed for the reaction. Under such the conditions thickening of iron is really high—a solid residue contains approximately 95% (*m*/*m*) of iron oxides and less than 1% of zinc—but selective recovery of zinc is very problematic. Hot sulfuric acid dissolves both zinc and iron (III) oxides to the similar extent producing a solution of highly concentrated zinc and iron (III) sulfates (VI). In the proposed method directing the prepared aqueous solution of SO_2_ onto the previously roasted material depleted of sulfur to the highest possible extent results in the formation of zinc and eventually iron bisulfates (IV).

Zinc present in the acidified solution in the cationic form can be separated from the solution using a saturated solution of sodium carbonate (Na_2_CO_3_). Adding portions of a saturated solution of Na_2_CO_3_ to an aqueous solution of Zn(HSO_3_)_2_ or ZnSO_4_ results in the neutralization of the acidic leaching solution, and, subsequently, precipitation of white-gray precipitate of basic zinc carbonate ([ZnCO_3_]_2_[Zn(OH)_2_]_3_) from the solution. The percentage of zinc in this compound is 59.5%. This value may be a useful criterion of the level of the impurities in the precipitate.

**Figure 4:**
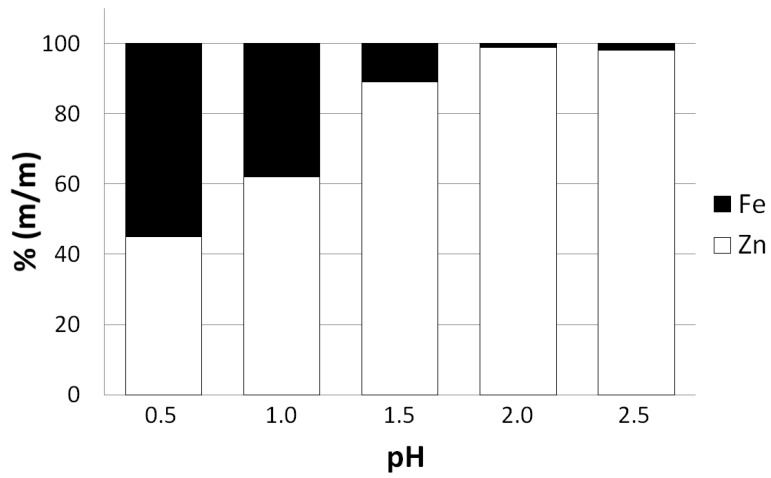
Mass fractions of iron and zinc (100 *m_i_*/(*m_Fe_* + *m_Zn_*), *i* = Fe, Zn) determined for the basic zinc carbonate (final product of the waste material processing) as a function of pH of leaching solution.

It is worth mentioning that reducing pH of the leaching solution below the recommended value of 2 significantly lowers the selectivity of the zinc leaching process (Figure 4). The obtained precipitate of basic zinc carbonate may be contaminated by iron in a form of iron hydroxide and carbonate and other metals due to their adsorption on the particles of iron hydroxide. Figure 4 reveals that for the lowest pH of the leaching solution employed in the experimental examination (pH = 0.5) iron mass fraction may be comparable to zinc mass fraction in the obtained zinc precipitate.

Lowering pH or directly saturating the suspension of the roasted material with SO_2_ also leads to the intensification of the leaching of calcium from its compounds present in the zinc-ferric material and results in an increased content of calcium carbonate (CaCO_3_) in the obtained precipitate of zinc salt. However, from the perspective of the subsequent stages of pyrometallurgical method of obtaining metallic zinc, the presence of calcium carbonate is advantageous, as its thermal decomposition in the presence of carbon improves the reducing abilities of the atmosphere in zinc smelting.

The gray-white precipitate of basic zinc carbonate, obtained under the suggested leaching conditions in the laboratory tests, is of at least 90% purity. The applied procedure made it possible to recover approximately 40% of the initial zinc content of the tested material.

The final stage of the proposed procedure involves converting the solid residue after acid leaching of zinc into a suspension and subjecting it to an external magnetic field in wet magnetic separators. The process of wet magnetic separation allows to thicken the iron oxides to a level of 55% (*m*/*m*). The goal of this step is to obtain material whose chemical properties (i.e., chemical composition in terms of quality and quantity) are as close as possible to the requirements for a blast furnace charge. The process of wet magnetic separation allows one to obtain an iron oxide fraction of the material (whose weight was equal to at least 1/3 of the initial weight of the sample subjected to leaching) contaminated by less than 12% of SiO_2_, 10% of CaO, 1.5% of Zn and 1.5% of other metals. These parameters allow the obtained material to be classified as a good substitute for iron ore.

### 3.1. Influence of the Presence of Iron and Lead Compounds on the Zinc Leaching Efficiency

The formation of iron (III) bisulfate (IV) is a competitive reaction that occurs when the aqueous suspension of a material is being directly saturated with gaseous SO_2_ according to the following chemical equation

Fe_2_O_3_ + 3H_2_O + 6SO_2_ → 2Fe(HSO_3_)_3_. (6)

The efficiency of this reaction intensifies also when the pH of the suspension reaches values below 2. Therefore, the leaching of iron can be reduced by using an acidic solution of pH not lower than 2. This enables the conversion of mainly zinc oxide to the soluble bisulfate (IV) (Zn(HSO_3_)_2_) form. If the leaching is carried out with diluted sulfuric acid (VI), zinc oxide is dissolved and a highly water-soluble zinc sulfate (VI) (ZnSO_4_) is formed.

Repetitive rinsing of the zinc-containing material with an aqueous solution of SO_2_ of pH not lower than 2 or with a solution of sulfuric acid (VI) at a concentration of 0.1%–0.5% enables complete, almost quantitative leaching of zinc oxide. The resulting filtrate is also free of lead compounds. The oxide forms of lead present in the roasted material are not leached by the formed sulfuric acid (IV) due to the practically insolubility of lead (II) bisulfate (IV) (Pb(HSO_3_)_2_) or lead (II) sulfate (VI) (PbSO_4_) in water.

### 3.2. Influence of the Presence of Calcium Compounds on the Zinc Leaching Efficiency

An increased content of calcium compounds in the processed material also reduces the efficiency of the zinc leaching process. The is due to the occurrence of a competitive reaction of formation of calcium bisulfate (IV) according to the following formula:

CaO + H_2_O + 2SO_2_ → Ca(HSO_3_)_2_. (7)

After the roasting process calcium is predominately represented by CaO which makes an aqueous suspension of the roasted oxide material slightly basic (pH in the range of 7.5–8.0).

With a high content of calcium compounds in the processed material, the majority of the generated SO_2_ is used to neutralize the solution with the formation of calcium bisulfate (IV). This, at the CaO content of 6% and above, hinders the leaching of zinc from the material to such an extent that the entire process takes a long time and consumes great amounts of SO_2_ and water. Therefore, it is desirable, at a limited supply of SO_2_, to neutralize the aqueous suspension of the roasted material with a 2% solution of sulfuric acid (VI) if the content of CaO is above 6%.

The computer program Medusa (by I. Puigdomenech) was used to estimate roughly the distribution of various forms of Zn, Fe, and Ca in a function of the solution pH. The simulation results are presented in Figures S1–S4 for ionic distributions of SO_3_^2−^, Zn^2+^, Fe^3+^, and Ca^2+^, respectively in forms of chemical equilibrium diagrams. The obtained data confirm that the selected pH value of the leaching solution is optimal for the selective extraction of Zn in the presence of Fe_2_O_3_ and Ca^2+^ cations.

## 4. Conclusions

The proposed method of processing of sulfur-bearing zinc-ferric materials results in a significant (up to 40%) reduction of the zinc content in the raw material with simultaneous generation of a fraction of material with substantially higher content of iron oxides. The selection of the adequate pH of the aqueous medium in which the leaching process is carried out is the key factor determining the quantitative and selective separation of zinc.

An important requirement of the developed method is the presence of sulfur in the processed material. If the amount of sulfur is insufficient, it may be supplemented by adding sulfuric acid (VI) to adjust the pH. An alternative approach involves using a leaching bath that consists of sulfuric acid (VI) solution at a concentration of 0.1%–0.5%.

The efficiency of iron thickening can be increased by employing the external magnetic field which, at the beginning of the material processing, roughly eliminates the ferromagnetic fraction consisting of iron oxide particles.

The Zn recovery efficiency was determined on the basis of XRF results obtained for the powdered raw materials, residues, and precipitates. The results of the elemental analyses were produced by the validated XRF method capable of analyzing quantitatively diverse samples [13].

## 5. Patents

A patent application on thickening iron and recovery of zinc from waste materials from processing zinc and lead ores has been filed by Greenmet Ltd. with W. Hyk, D. Rudnicki and K. Kitka as inventors. The current status of this application: withdrawn.

## Figures and Tables

**Table 1:** X-ray Fluorescence (XRF) determinations (% (*m*/*m*)) of chemical composition of test samples representing the three selected sections of stockpiled waste materials. The numbers in brackets represent the metal determinations by an Inductively Coupled Plasma Mass Spectrometer (ICP-MS). The relative expanded uncertainties of the XRF and ICP-MS results are 18% and 8%, respectively.

Sample No.	Fe	Zn	Pb	S	SiO_2_	CaO	As	Cu
1	26.2 (25.2)	11.6 (13.1)	6.9 (4.7)	6.6	7.8	2.5	1.7 (1.7)	1.6 (1.7)
2	17.4	4.0	1.2	1.5	10.1	15.9	0.3	0.5
3	22.9	7.3	2.6	4.1	9.8	5.8	0.9	0.2

## Supplementary Materials

The following are available online, Figure S1: Chemical equilibrium diagram for 10 mM SO_3_^2−^ in a function of the solution pH. Figure S2: Chemical equilibrium diagram for 10 mM Zn^2+^ in a function of the solution pH. Figure S3: Chemical equilibrium diagram for 10 mM Fe^3+^ in a function of the solution pH. Figure S4: Chemical equilibrium diagram for 10 mM Ca^2+^ in a function of the solution pH.
