# Supplementary material for: Selective Recovery of Zinc from Metallurgical Waste Materials from Processing Zinc and Lead Ores
Source: Molecules. 2019 Jun 19;24(12):2275. doi: 10.3390/molecules24122275 (PMC6631940; doi:10.3390/molecules24122275)
Supplement: Supplementary file 1 [file molecules-24-02275-s001.pdf]

Supplementary Materials

Selective recovery of zinc from metallurgical waste materials from processing zinc and lead ores

Wojciech Hyk [1,2,*], Konrad Kitka [1,2] and Dariusz Rudnicki [3]

1 Faculty of Chemistry, University of Warsaw, Pasteura 1, PL-02-093 Warsaw, Poland
2 Faculty of Chemistry, Biological and Chemical Research Center, University of Warsaw, Żwirki i Wigury 101, PL-02-089 Warsaw, Poland
3 Greenmet Technology Ltd., Chełmońskiego 67, PL-96-321 Żelechów, Poland

* Correspondence: wojhyk@chem.uw.edu.pl; Tel.: +48-22 5526359

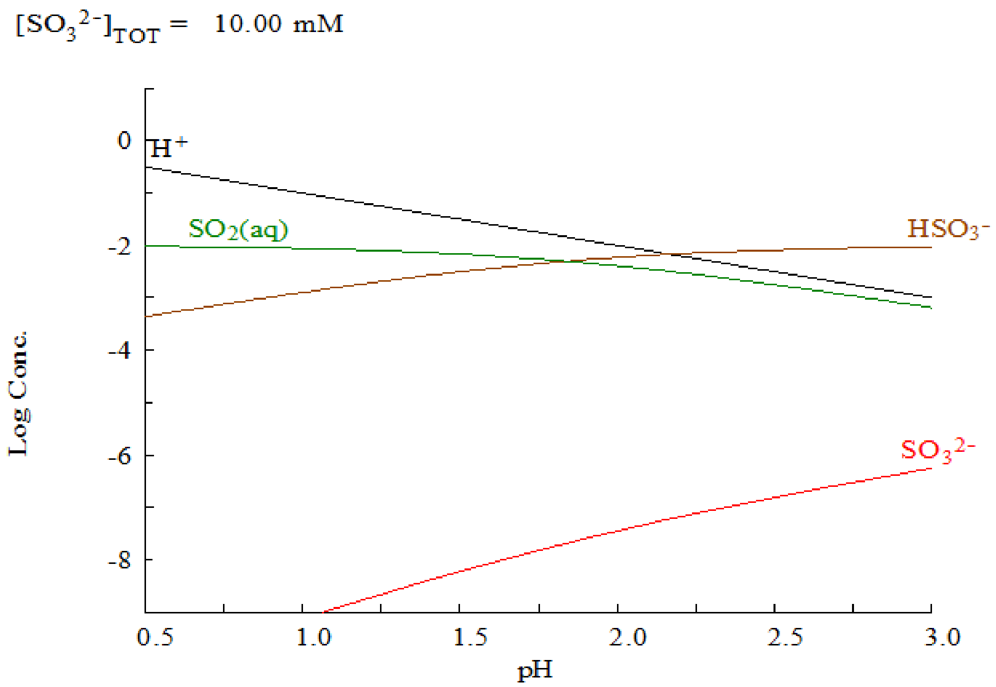

Figure S1. Chemical equilibrium diagram for 10 mM SO_3^{2-} in a function of the solution pH.

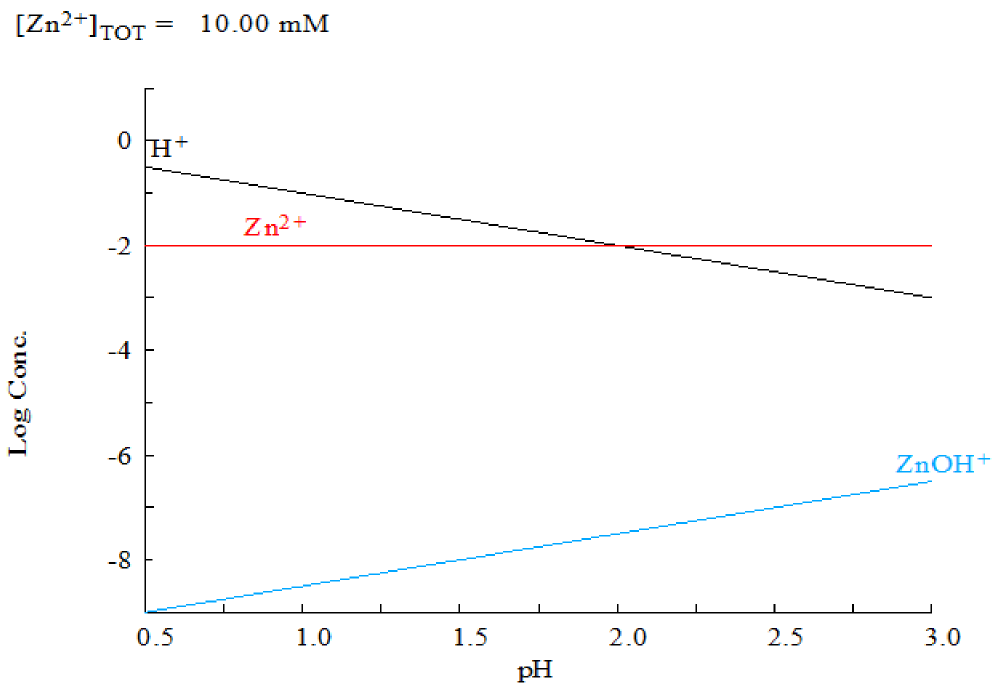

Figure S2. Chemical equilibrium diagram for 10 mM Zn^{2+} in a function of the solution pH.

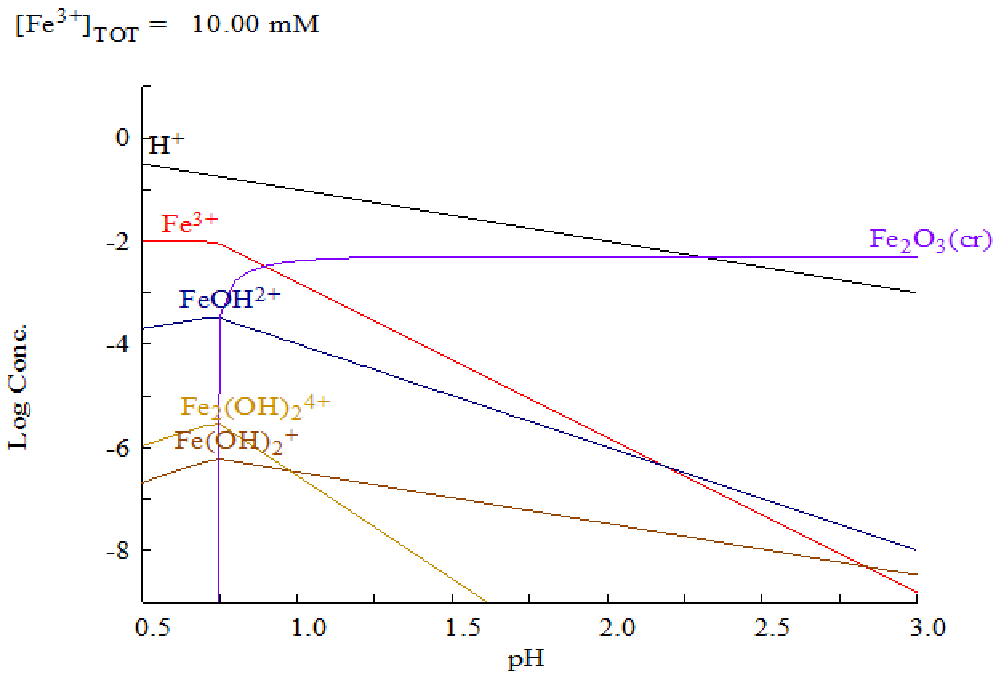

Figure S3. Chemical equilibrium diagram for 10 mM Fe^{3+} in a function of the solution pH.

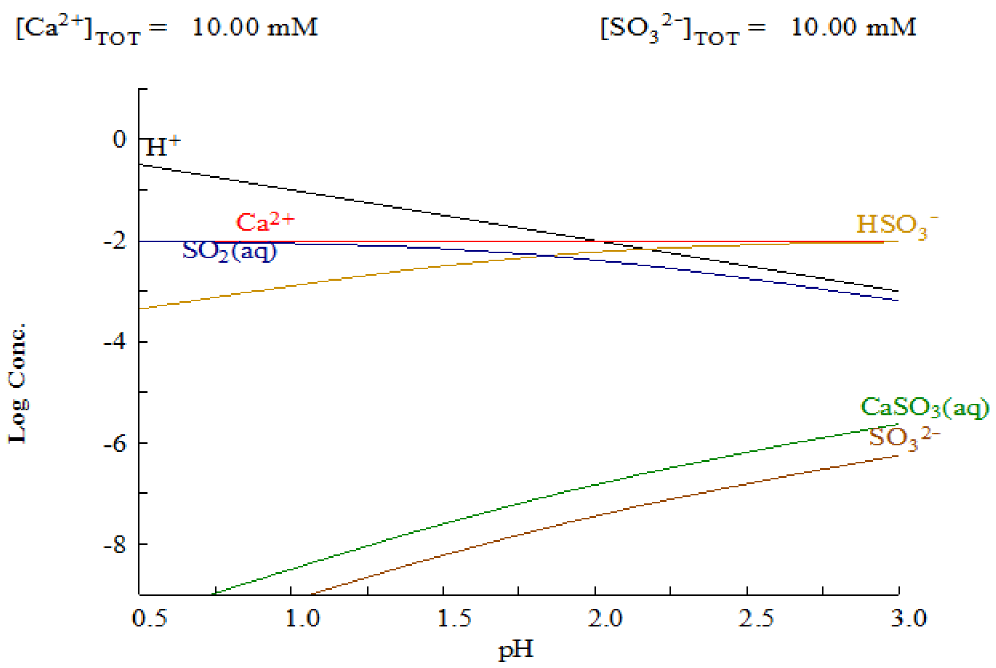

Figure S4. Chemical equilibrium diagram for 10 mM Ca^{2+} in a function of the solution pH.
